# Phylogenetic Integration Reveals the Zebrafish Core Microbiome and Its Sensitivity to Environmental Exposures

**DOI:** 10.3390/toxics9010010

**Published:** 2021-01-15

**Authors:** Thomas J. Sharpton, Keaton Stagaman, Michael J. Sieler, Holly K. Arnold, Edward W. Davis

**Affiliations:** 1 Department of Microbiology, Oregon State University, Corvallis, OR 97331, USA; stagamak@oregonstate.edu (K.S.); sielerjm@oregonstate.edu (M.J.S.J.); arnoldho@oregonstate.edu (H.K.A.); 2 Department of Statistics, Oregon State University, Corvallis, OR 97331, USA; 3 Center for Genome Research and Biocomputing, Oregon State University, Corvallis, OR 97331, USA; ed@cgrb.oregonstate.edu; 4 Carlson College of Veterinary Medicine, Oregon State University, Corvallis, OR 97331, USA

**Keywords:** zebrafish, gut microbiome, environmental exposure, phylogenetics, meta-analysis

## Abstract

Zebrafish are increasingly used to study how environmental exposures impact vertebrate gut microbes. However, we understand little about which microbial taxa are common to the zebrafish gut across studies and facilities. Here, we define the zebrafish core gut microbiome to resolve microbiota that are both relatively robust to study or facility effects and likely to drive proper microbiome assembly and functioning due to their conservation. To do so, we integrated publicly available gut microbiome 16S gene sequence data from eight studies into a phylogeny and identified monophyletic clades of gut bacteria that are unexpectedly prevalent across individuals. Doing so revealed 585 core clades of bacteria in the zebrafish gut, including clades within *Aeromonas*, *Pseudomonas*, *Cetobacterium*, *Shewanella*, *Chitinibacter*, *Fluviicola*, *Flectobacillus*, and *Paucibacter*. We then applied linear regression to discern which of these core clades are sensitive to an array of different environmental exposures. We found that 200 core clades were insensitive to any exposure we assessed, while 134 core clades were sensitive to more than two exposures. Overall, our analysis defines the zebrafish core gut microbiome and its sensitivity to exposure, which helps future studies to assess the robustness of their results and prioritize taxa for empirical assessments of how gut microbiota mediate the effects of exposure on the zebrafish host.

## 1. Introduction

The zebrafish has emerged as a powerful model for studying the interactions between a vertebrate’s environment, gut microbiome, and physiology [1]. For example, a rapidly growing number of investigations have leveraged zebrafish to determine how exposure to exogenous factors, including antibiotics [2,3,4], environmental toxicants [5,6], or parasites [7], impacts the composition of the juvenile or adult gut microbiome. Additionally, studies have also resolved microbiota that link to host physiology [8,9,10] as well as gut taxa that appear to mediate the impact of various exposures on physiology [4,7,11]. It remains unclear, however, whether the interactions that have been uncovered to date are robust to study or facility effects, which can contribute substantial variation to microbiome studies [12,13]. This limited insight in turn complicates the interpretation and prioritization of microbiome study results, especially for follow-up investigations that seek to discover robust mechanisms of interaction.

One way to advance this knowledge is to determine which gut microbes constitute the zebrafish core gut microbiome. While there are many ways of defining the core gut microbiome [14,15], it typically comprises the set of taxa that are nearly ubiquitous across presumably healthy host individuals. In addition to clarifying which taxa are robust to facility and experimental effects, identifying the core taxa provides a framework for testing which taxa are critical to the assembly and function of a gut microbiome that provides the most beneficial contribution to host physiology [16]. While extensive investigations have sought to define the core gut microbiome in a variety of mammals, we understand little about whether zebrafish possess a core gut microbiome and, if they do, which taxa comprise it. That said, a prior investigation used clone libraries alongside a limited exploration of 454 sequencing to uncover evidence that pointed to the existence of a zebrafish core gut microbiome [17]. Moreover, zebrafish studies often identify consistent phylotypes in the gut that are linked to exposure or health, such as *Pseudomonas*, *Aeromonas*, and *Vibrio* [2,6,7,18,19]. Inspired by this prior work and the growth of zebrafish gut microbiome studies, we sought to empirically define which taxa comprise the zebrafish core gut microbiome and measure their sensitivity to various exposures. 

Identifying which taxa comprise the gut microbiome is not necessarily straightforward. Frequently, studies will identify core taxa by measuring the prevalence of different phylotypes across a set of microbiome samples. Core taxa are then defined as those phylotypes that meet some threshold of prevalence. This phylotype approach yields at least two forms of bias that can complicate the identification of core taxa: (1) the prevalence threshold is arbitrarily defined; and (2) Linnaean taxonomy, which serves as the basis for phylotypes, is inconsistent across the tree of life and may obscure resolutions of evolutionarily meaningful, but cryptic, core sub-taxa [20]. We have developed an alternative approach that leverages microbial phylogenetics to circumvent these biases [20]. Briefly, our approach assembles a phylogeny of the 16S rRNA gene sequences of the taxa observed in each microbiome sample and resolves specific monophyletic clades in this phylogeny whose prevalence across all samples is higher than expected by chance given the clade’s size. As a result, this approach considers intermediate levels of taxonomy as defined by the phylogeny, and leverages a statistical test to ascertain which taxonomic groups are sufficiently prevalent to be considered part of the core microbiome. 

Here, we used this phylogenetic approach to define the zebrafish core gut microbiome and its sensitivity to various environmental exposures. In particular, we assembled a phylogeny that integrated publicly available 16S rRNA gene sequence data generated from eight different zebrafish gut microbiome studies that span an array of facilities, ages, and exposure conditions. We then used our procedure to identify monophyletic clades that were more prevalent in unexposed fish across these studies than expected by chance, which we defined to collectively constitute the core zebrafish gut microbiome. Finally, we used linear regression to profile the sensitivity of these clades across a range of exposure conditions.

## 2. Materials and Methods 

### 2.1. Data Acquisition and Study Inclusion Criteria

Studies for our integrative analysis were identified through a literature search of Google Scholar and NCBI PubMed. To be included in the analysis, all studies were required to have: (1) been sampled from zebrafish (*Danio rerio*); (2) used high-throughput sequencing of the 16S rRNA gene; (3) associated metadata; and (4) data that were freely available. Sequence files from each study were downloaded from the short read archive (SRA) or provided by study investigators. A total of 8 studies met our inclusion criteria (Table 1).

### 2.2. S rRNA Gene Sequence Quality Control and Amplicon Sequence Variant Clustering

Quality control of 16S sequences and amplicon sequence variant (ASV) calling was conducted on a per-project basis using a standard dada2 (v1.16.0) [21] pipeline in R (v4.0.3) [22]. For each dataset, forward and reverse reads containing ambiguous base calls or an expected error rate greater than 2 were dropped. For projects with raw read lengths greater than 150, forward reads were trimmed to 225 bp and reverse reads trimmed to 150 bp, and then forward and reverse reads were merged. The pipeline included chimera removal, and assigned taxonomy to ASVs using the Silva NR v138 training database [23]. ASVs assigned to Eukarya were discarded. ASV abundance tables were then rarefied to 1000 sequences per sample to ensure uniform sequencing depth across studies, and the sequences of the ASVs that survived rarefaction were subject to the subsequent phylogenetic analyses. Assessment of ASV beta-diversity was determined using the phyloseq (v1.32.0) library in R [24]. In particular, weighted and unweighted unifrac statistics were quantified, subject to ordination using principal coordinates analysis (PCoA), and statistically associated with sample covariates through PERMANOVA tests as implemented in the *adonis* function through the vegan (v2.5.6) package [25]. These phylogenetic-based beta-diversity metrics were chosen to account for taxa that, because of our analytical pipeline, were assigned differing ASV IDs, but may actually be closely related or even the same strain. To facilitate measuring ASV prevalence across studies, ASV sequences were directly compared and those that were either identical or perfect subsets of other ASV sequences were agglomerated into a representative ASV. The observations of these identical ASVs were also agglomerated to profile the representative ASV’s presence and abundance across samples.

### 2.3. Phylogenetic Analysis

Following our prior work [20], we assembled a phylogenetic tree relating the rarefied ASV sequences identified across the studies. Briefly, the 4195 ASV sequences were aligned to the SILVA (v1.2.3) reference alignment using the mothur (v1.39.3) implementation of the NAST aligner [26] with the setting flip = TRUE. We included 100 full-length, phylogenetically diverse guide sequences in this alignment to improve the accuracy of phylogenetic reconstruction [20,27]. The resulting alignment was then subject to phylogenetic inference using FastTree (v2.1.10) with the settings -nt -gtr [28]. Custom R scripts (v4.0.3) that invoked the ape package (v5.4.1) function *drop.tips* pruned guide sequences from the phylogeny to produce a phylogenetic tree relating to study ASVs [29]. This tree was then subject to midpoint rooting using the *midpoint.root* function in the phytools (v0.7.70) R package [30]. The resulting phylogeny data are included in the Supplementary Materials.

### 2.4. Ecophylogenetic Discovery of Core Clades

We used the ClaaTU algorithm [20] to identify clades that are more prevalent in the control samples across studies than expected by chance. The ClaaTU algorithm traverses a phylogenetic tree relating to ASV sequences and, for each monophyletic clade found in the tree, sums the abundance of each subtending lineage found in each sample. These sums represent the clade’s abundance in each sample, akin to the process of agglomerating ASV abundances to discern phylotype abundance. ClaaTU then measures the prevalence of each clade in each sample and quantifies if the observed prevalence is significantly greater than that expected by chance. To do so, it first bootstraps a null distribution of each clade’s prevalence across samples by randomly permuting the association between tips in the phylogeny and ASV labels. A z-test then determines if each clade’s observed prevalence significantly differs from its null distribution. Clades exhibiting a significantly greater prevalence were deemed to be core components of the gut microbiome. In particular, we identified clades that were core to the control samples (i.e., no exposure) across studies irrespective of fish age, facility, or strain. To produce null distributions of clade prevalence, we permutated associations between trees and ASVs 999 times. Core clades were identified as those whose prevalence across samples was significantly greater than the null distribution when using a false discovery corrected *p*-value threshold of 0.05 (i.e., *p* < 0.05). The resulting clade diversity matrix, clade prevalence values, and statistical test results are included as supplemental data (Supplementary Table S1).

### 2.5. Linear Regression

We implemented negative binomial linear regression models to determine whether the abundance of core clades significantly varied across samples as a function of specific exposures. Negative binomial distributions are appropriate for modeling the sparse and over-dispersed count data typically observed in microbiome experiments [7,31]. In particular, we used the glm.nb function in the MASS (v7.3.53) R package to construct distinct models for each study, because studies included non-overlapping and sometimes multiple exposures [32]. For each study, we regressed each core clade’s abundance in a sample as a function of that sample’s exposure condition. In particular, the clade abundance was measured as the agglomerated clade abundances produced by ClaaTU, and the exposure type is the different exposure categories applied in a given study, including the category of no exposure (i.e., control samples), which was set as the base level in each model. Clades for which a given exposure type’s model coefficient was significantly different than that of the unexposed condition using a false discovery rate (fdr) corrected threshold of 0.1 (i.e., fdr < 0.1) were determined to be sensitive to the exposure. 

## 3. Results

### 3.1. Study and Age Effects Drive the Phylogenetic Composition of the Zebrafish Gut Microbiome 

To uncover the core zebrafish gut microbiome, we downloaded publicly available 16S rRNA gene sequence data from eight studies spanning five different zebrafish facilities. The collective dataset included 582 samples, 316 of which were subject to one of nine different environmental exposure conditions. We then applied a consistent informatic approach to each study’s data to resolve amplicon sequence variants (ASVs) within each study and measured their abundance across samples. In total, our analysis identified 3814 distinct ASVs spanning this set of samples after rarefaction. ASVs ranged from 225 to 254 bp in length.

**Figure 1:**
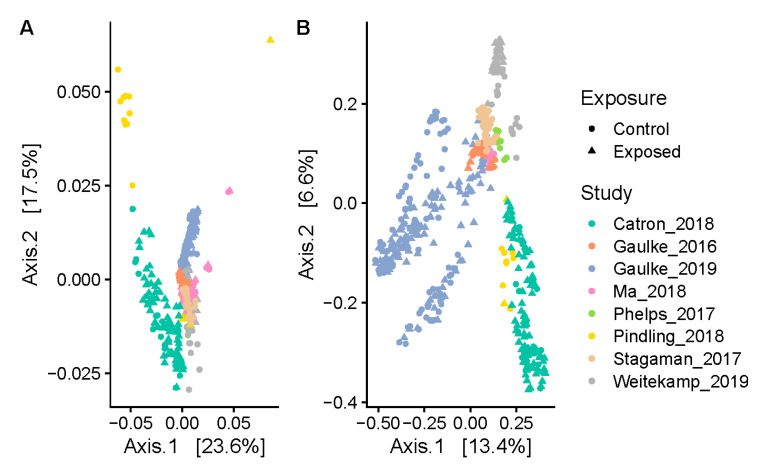
The phylogenetic composition of the zebrafish gut microbiome varies by study. These principle coordinate plots (PCoAs) illustrate the (**A**) weighted and (**B**) unweighted unifrac distance between all samples based on their amplicon sequence variant (ASV) profiles.

We first measured the extent to which experiment, facility, age, and exposure effects impacted the distribution of ASVs across individuals (Figure 1). To do so, we quantified the phylogenetic beta-diversity across samples using the weighted and unweighted unifrac distance metrics. These metrics were used because they leverage phylogenetic distances between ASVs to inform on the similarity between the composition of two communities, which was critical in our study because ASVs may appear to be distinct—albeit phylogenetically proximal—as a result of technical differences across studies. PERMANOVA tests revealed that the phylogenetic composition of the zebrafish gut microbiome is dominated by study effects (Weighted Unifrac: R^2^ = 0.326, *p* < 0.001; Unweighted Unifrac: R^2^ = 0.264, *p* < 0.001). Facility effects also play a role (Weighted Unifrac: R^2^ = 0.193, *p* < 0.001; Unweighted Unifrac: R^2^ = 0.192, *p* < 0.001), but are generally confounded with study effects. Moreover, the phylogenetic composition of the gut microbiome also significantly differed across fish based on their age (Weighted Unifrac: R^2^ = 0.216, *p* < 0.001; Unweighted Unifrac: R^2^ = 0.247, *p* < 0.01). We also stratified samples by their study of origin and assessed if samples subject to an exposure are generally distinct from their paired controls. We found that exposed samples weakly differentiate from controls when considering the presence of phylogenetic lineages (Unweighted Unifrac: R^2^ = 0.0178, *p* < 0.001), but not when considering the abundance of phylogenetic lineages (Weighted Unifrac: R^2^ = 0.022, *p* = 0.194). This result indicates that although exposure may affect which taxa are present in the gut, simply being exposed to an exogenous factor may not consistently result in a different set of dominant taxa in the gut as compared to unexposed controls. Rather, as found in prior work [6], specific exposures may elicit specific effects on which taxa dominate the zebrafish gut microbiome.

### 3.2. A Small Set of ASVs Are Common to Zebrafish across Studies

**Figure 2:**
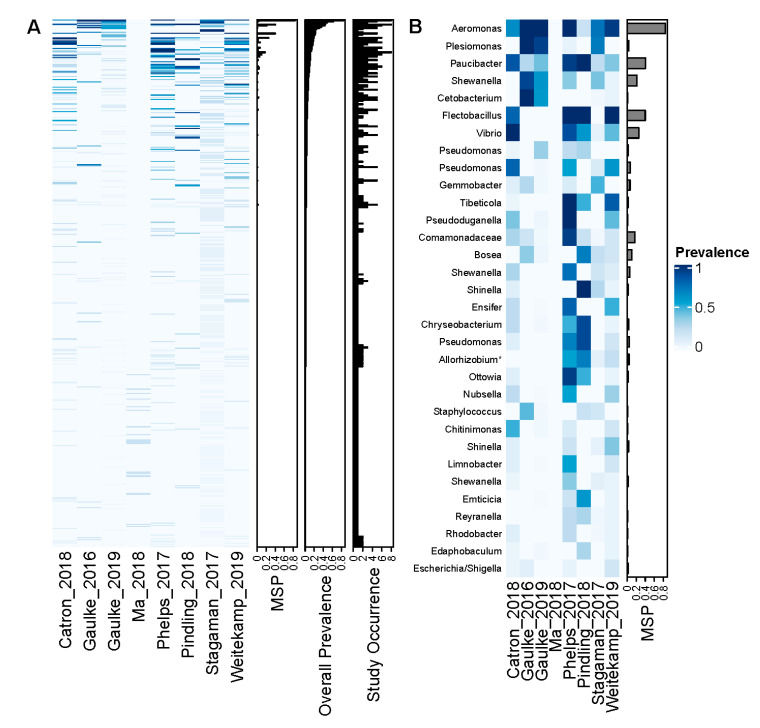
A small number of ASVs occur across multiple studies after merging ASVs that are identical or perfect substrings of other ASVs across studies. Both panels illustrate heatmaps that represent specific ASVs as rows and studies as columns. Panel (**A**) shows the prevalence of all ASVs detected in our analysis, ordered by their overall prevalence. Row bar plots illustrate the median prevalence per study (MSP), the overall prevalence, and whether the ASV occurs at least one time in at least one individual within a study (study occurrence). Panel (**B**) illustrates the same data but zooms in on the ASVs that are present in at least 5 studies. Row names correspond to the genus-level annotation of the ASV (or family in the case of an ambiguous genus-level annotation).

We then quantified the prevalence of ASVs across all studies as well as within each study to ascertain if core microbiota may exist at the ASV level. While we clustered ASVs independently per project to optimize error learning, we informatically merged ASVs based on sequence identity to resolve ASVs that were prevalent across studies. We quantified the prevalence of these ASVs across the 266 control (“unexposed”) samples to prevent exposure effects from spuriously decreasing the apparent prevalence of ASVs. As illustrated in Figure 2, when assessing the prevalence of each ASV across control samples irrespective of their study of origin, we find that each ASV is, on average, present in 0.62% of these samples (median = 0.38%). The 477 ASVs in the top quartile of sample prevalence manifested prevalence rates ranged from 0.38% to 84.2%. While the vast majority of these relatively high prevalence ASVs were present in a single study, 130 were found in more than one study, and 32 were found in more than three studies. Of them, seven ASVs appeared to be extremely prolific, because they appeared in more than half of the studies and their median per study prevalence (MSP) exceeded 10%. These ASVs were members of Aeromonas (MSP = 84.3%; study number = 8), Paucibacter (MSP = 40.5%; study number = 8), Flectobacillus (MSP = 40.0%, study number = 5), Vibrio (MSP = 25.4%; study number = 6), Shewanella (MSP = 21.5%; study number = 6), an ASV within the Comamonadaceae (MSP = 17.4%, study number = 8), and Bosea (MSP = 10.0%, study number = 6). Collectively, these results indicate that although the vast majority of observed zebrafish gut ASVs are not common across individuals, a small number of ASVs appear to be relatively prolific. However, it is challenging to ascertain which ASVs constitute core taxa given the arbitrary thresholds applied above. Moreover, the fine-scale taxonomic resolution of ASVs may obscure detection of prevalent taxonomic units, which in turn results in an underestimated size of the core zebrafish gut microbiome. 

### 3.3. A Diverse Set of Clades Comprise the Core Zebrafish Gut Microbiome

**Figure 3:**
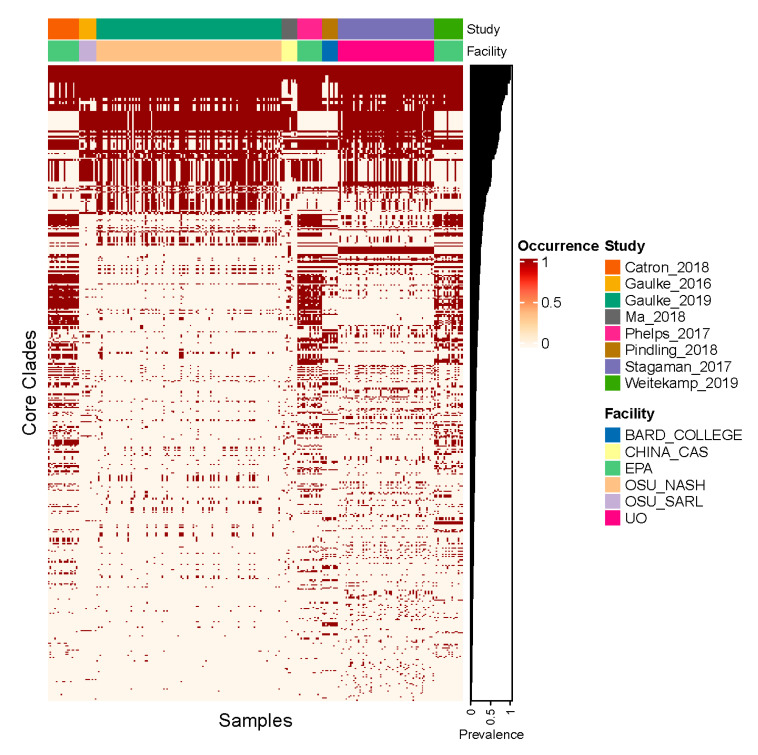
Core clade prevalence across all samples. In this heatmap, columns correspond to the specific samples evaluated in our analysis, grouped by study, and the rows correspond to each of the core clades detected in our investigation, grouped by their overall prevalence across samples.

We next investigated whether core monophyletic clades of ASVs existed in the zebrafish gut, which we defined as clades whose prevalence across samples was significantly greater than expected by chance. As with the ASV analysis, we limited our consideration of samples to the 266 that were not subject to experimental exposures. In so doing, we identified 585 core clades of ASVs out of the 4054 total clades present in the phylogeny we assembled. As illustrated in Figure 3, the prevalence of these clades across samples irrespective of their study of origin ranged from 0.75–100%, and the median prevalence was 12.5%. The low prevalence values for some of these clades can be attributed to their very recent divergence, because the average tip-to-tip phylogenetic distance between lineages within a clade correlates with overall prevalence across samples (Spearman’s rho = 0.472, *p* < 0.001). While these clades manifest low prevalence rates, they were more prevalent than expected by chance given the clade’s size. Moreover, these clades tend to be prevalent across multiple studies; 53% of these core clades (313 clades) were present in four or more studies. 

**Figure 4:**
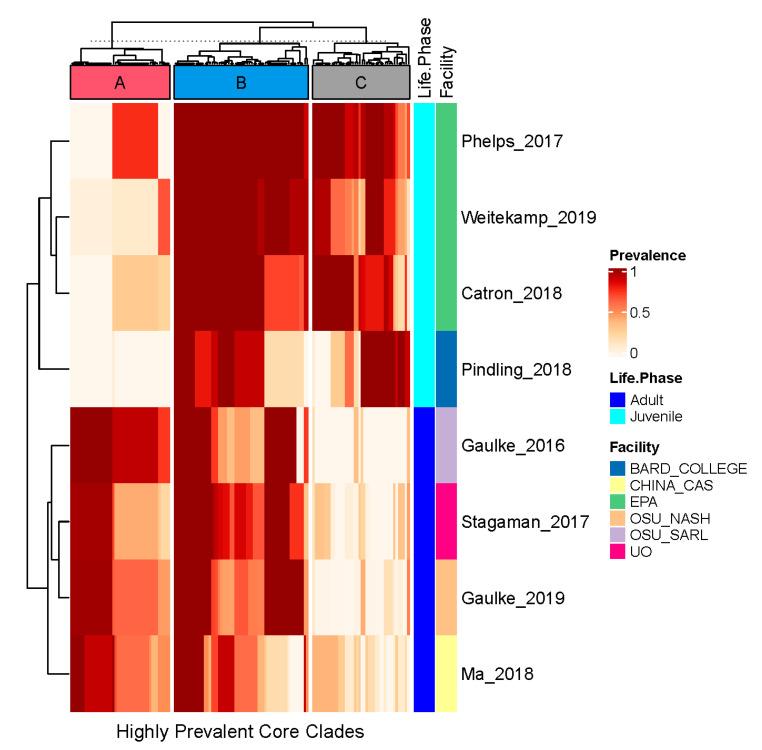
The study-wide prevalence of the top 25% most prevalent clades. In this heatmap, columns correspond to the top 25% most prevalent core clades. Rows correspond to a particular study, and the cells illustrate the prevalence of the clade across the samples evaluated within each study. Hierarchical clustering and kmeans clustering support the existence of three groups of highly prevalent clades that are generally life stage-specific or robust to life stage.

To better understand high-prevalence core clades, we assessed how the top 25% most prevalent clades were distributed across studies. As shown in Figure 4, hierarchical clustering of each of these 143 most prevalent clades reveals that the clades are grouped into three major clusters (A, B, C) based on their prevalence distribution across samples. This grouping is supported by kmeans clustering (centers = 3). These clusters appear to be defined by the clades’ prevalence across studies based on the age of the fish subject to investigation. For example, cluster A contains clades that are prevalent in adult fish and typically missing from juvenile fish. Cluster B contains clades that are effectively ubiquitous across all fish. Cluster C contains clades that are prevalent in juvenile fish and typically missing from adult fish. In general, these life phase effects appear to overwhelm study effects with respect to the prevalence distribution of these highly prevalent core clades. The taxonomy associated with all core clades, including these highly prevalent core clades, as well as their overall and median per study prevalence rates, is included as supplemental data (Supplementary Table S1).

### 3.4. Core Clades Differentially Respond to Environmental Exposures

**Figure 5:**
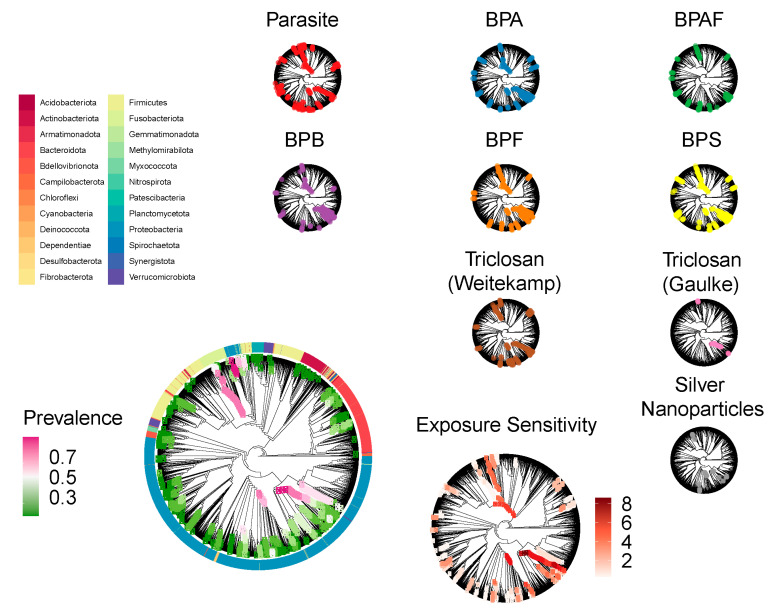
The zebrafish core gut microbiome is phylogenetically diverse. The above cladograms illustrate the phylogenetic relationship of the ASVs detected across all samples subject to our analysis. All cladograms are identical in topology and represent the same set of ASVs, but differ in the information that associates with cladogram decoration. The lower left cladogram illustrates the phylogenetic location of each of the core clades detected in our analysis, where node color corresponds to the overall prevalence of each core clade. The ring of colors encircling the cladogram illustrates each ASV’s phylum level taxonomy. The clade to the immediate right illustrates the number of exposures to which each of these core clades is sensitive, as measured by negative binomial regression models. The smaller cladograms near the top illustrate which clades are sensitive to specific exposures (fdr < 0.1).

**Figure 6:**
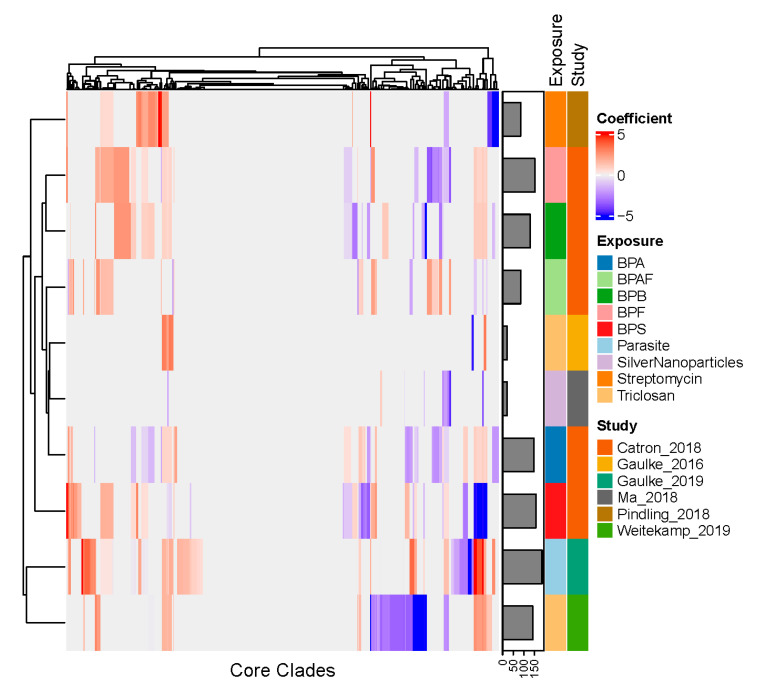
Core clades differentially respond to environmental exposures. In this heatmap, zebrafish gut microbiome core clades are represented as columns and exposure states included in different studies are represented as rows. Heatmap colors represent negative binomial model regression coefficients of the clade’s count within a rarefied sample as a function of the exposure. Both columns and rows were subject to hierarchical clustering and organized accordingly in the heatmap, as illustrated by the dendrograms decorating the top and left side of the heatmap. The bar plot to the right of the heatmap illustrates the number of core clades that were sensitive to the exposure in question (fdr < 0.1).

We next sought to clarify how different environmental exposures impact the zebrafish core gut microbiome (Figure 5). To do so, we used negative binomial regression to model each core clade’s abundance across the samples from each study as a function of the different exposure conditions applied in that study. As illustrated in Figure 6, core clades vary in their profile of sensitivity across exposure conditions. In particular, 385 core clades were sensitive to at least one exposure, while the remaining 200 clades appeared to be robust to the exposures used in the studies considered here (fdr < 0.1). Of these sensitive clades, 134 were sensitive to at least three different exposures, placing them in the top quartile of exposure sensitivity. These clades inconsistently responded to exposures, such that differences in a clade’s relative abundance as compared to unexposed controls increased in response to some exposures, while it decreased in response to others. For example, the core clades that increased the most in abundance, which resulted from exposure to P. tomentosa, were also among the set that decreased the most in abundance, which resulted from exposure to the chemical BPS. These observations indicate that while some core clades appear to be robust to exposure, others elicit extensive variation in their response to exposures. 

Additionally, exposures vary considerably in their effect on core clades. Exposure to parasites in adults affects the largest number of core clades (186 each), while exposure to triclosan or silver nanoparticles affects the smallest number of core clades (21 clades each). The impact of exposure on core clades does not appear to be driven by life stage, study, or facility, as evidenced by the hierarchical clustering. For example, Catron et al. [6] exposed juvenile fish to five different BPA metabolites using the same facility. These metabolites varied widely in terms of the number of core clades they affected (range 86–158) and clustered distinctly from one another based on the specific clades they impacted. However, triclosan, the only exposure included in more than one study, elicited substantially different effects on core clades in the adult zebrafish studied by Gaulke et al. [2] (21 affected core clades) as compared to the juvenile fish studied by Weitekamp et al. [4] (142 affected core clades), suggesting that age (or alternatively differences in the route or concentration of exposure) may mediate how specific exposure types affect the gut microbiome. Collectively, these results indicate that the core gut microbiome’s sensitivity to exposure may be largely driven by specific exposure parameters (e.g., exposure concentration, duration, etc.). 

## 4. Discussion

The identification of core microbiota has been a consistent, albeit somewhat elusive, objective in microbiome research. Driving the effort to identify such taxa is the assumption that, due to the fact that these taxa are more prevalent among healthy individuals than expected by chance given neutral processes, they are critical to the ecological functioning of the microbiome that promotes health. Putative core taxa can be used to generate hypotheses for researchers to experimentally test the microbiome’s impact on host physiology, and ultimately define biochemical or ecological mechanisms through which these host–microbiome interactions occur. Moreover, resolving core microbiota clarifies which taxa may comprise the minimum viable microbiome that associates with a healthy host, which advances efforts to develop model microbiomes and probiotic consortia. Finally, such taxa are valuable to identify because they clarify which discoveries in a given microbiome investigation are likely robust to facility and experimental effects.

The identification of core microbiota is especially important in widely used model systems, such as zebrafish. Zebrafish have emerged as a valuable resource for studying both how the gut microbiome impacts vertebrate physiology and mediates the effects of environmental exposures on the host [1]. However, we understand little about which aspects of the zebrafish gut microbiome are robust to experimental and facility effects, and whether healthy fish associate with common gut microbes. Prior work points to the existence of a zebrafish core microbiome [17], but to date it has not been explicitly defined. By phylogenetically integrating microbiome sequence data across a litany of zebrafish studies, we identified a diverse core gut microbiome in zebrafish that is robust to facility, experimental, age, strain, and diet effects. One of the benefits of our phylogenetic approach to defining the core gut microbiome is that it resolves periods in the evolution of zebrafish gut microbiota that resulted in the innovation of traits that underlie the prevalent distribution of taxa across zebrafish. Although we do not know what those traits are or whether they matter to zebrafish physiology, our efforts uncovered phylogenetic groups of ASVs that have presumably conserved the traits in question, which facilitates future discovery of these traits. Regardless, our observations help contextualize discoveries made in zebrafish gut microbiome experiments, and as discussed below, align with prior work in such that the core taxa identified here are commonly observed in zebrafish gut microbiome investigations.

In particular, 14% of the monophyletic clades present in our integrated phylogeny showed statistical evidence of being more prevalent across samples than expected by chance. These clades are members of a diverse range of phylotypes, highlighting the phylogenetic diversity of the zebrafish core gut microbiome. For example, the top 25% most prevalent clades are members of the Gammaproteobacteria, Fusobacteria, Bacteroidetes, and Alphaproteobacteria. Many of these clades appear to have recently diverged, such that they are members of genus-level phylotypes. Notably, several of these phylotypes were also identified as common zebrafish gut phylotypes in prior work [17], and have been linked to zebrafish physiology or overall microbiome composition elsewhere. For example, a clade within the genus *Aeromonas* (node2572) was present in 84.6% of samples. Specific molecules produced by *Aeromonas* strains have been shown to influence immune system activation and even kidney beta-cell expansion in larval zebrafish [9,33]. A clade within the genus *Cetobacterium* (node2632) was present in 74.4% of samples. Members of *Cetobacterium* have previously been shown to be enriched among adult zebrafish intestines [18] and are negatively associated with parasite burden in the zebrafish gut [7]. A clade within *Shewanella* (node1650) was present in 51.5% of samples. *Shewanella* species have been shown to prevent immune responses induced by other members of the zebrafish microbiome (e.g., *Vibrio*), even when *Shewanella* is in relatively low abundance [34]. Other highly prevalent yet recently diverged clades include members of *Chitinibacter* (node780, 37.2% prevalence), *Fluviicola* (node3491, 30.0% prevalence), *Flectobacillus* (node3426, 22.6% prevalence), and *Paucibacter* (node971, 44.0% prevalence). Finally, clades within the Pseudomonadales were also highly prevalent. For example, a clade within the genus *Pseudomonas* (node 1555) was present in 45.5% of samples, and more divergent clades within the order were present in 77.1% of samples (e.g., node1474). Members of the Pseudomonadales, including *Pseudomonas*, were shown to protect zebrafish from infection by the pathogen *Flavobacterium columnare* [35].

An emerging goal in environmental health science is to determine how environmental exposure impacts the gut microbiome. Our investigation reveals that core microbiota vary in their sensitivity to different exposures. For example, some core clades, such as a clade in the genus *Shewanella* (node1670, 43.3% prevalence) and a clade in the genus *Acinetobacter* (node1578, 35.7% prevalence) are robust to all exposures we assessed. On the other hand, certain clades were much more sensitive, including a clade within the Gammaproteobacteria (node1382, 80.5% prevalence, sensitive to all 9 exposures), a clade within the genus *Rheinheimera* (node1546, 18.8% prevalence, sensitive to seven exposures), and a clade within the Pseudomonadales (node1475, 77.1% prevalence, sensitive to seven exposures). It is not apparent from the data we could access if exposure-induced effects on the core microbiome yield robust changes in fish physiology. That said, exposures impacted core clades that are members of phylotypes that have been previously linked to physiology. For example, as noted above, members of Pseudomonadales can protect fish from pathogens. Moreover, prior work showed that *Rheinheimera* is enriched in conventionalized germ-free fish that manifest normal neurobehavior as compared to their germ-free counterparts and conventionally reared fish [10]. Future work should seek to measure the effect of perturbing core taxa on fish physiology and whether these effects are consistent across different types of exposures.

Exposures also varied in how extensively they impacted the gut microbiome. For example, exposure to the intestinal parasite *Pseudocapillaria tomentosa* affected the largest number of core clades (186 clades), while exposure to the consumer grade antibiotic triclosan (at least in adults) or silver nanoparticles affected the smallest number of core clades (21 clades each). As noted in our results, simply being exposed to an exogenous factor only modestly affects the phylogenetic composition of the gut microbiome. Collectively, these observations support the expectation that different types of exposures select for different gut microbiome compositions and do so with different magnitudes. 

In addition to the aforementioned core clades, our analysis resolves a small set of highly prevalent ASVs. We currently lack a statistical framework for assessing whether an ASV’s prevalence is greater than that expected by chance; thus, studies frequently use conservative but arbitrary prevalence thresholds to identify core ASVs (e.g., 80% prevalence). We identified seven ASVs that were at least 10% prevalent across at least half of the studies we evaluated. Although the prevalence of these ASVs may not meet thresholds commonly employed to determine core taxa, the fact that these highly resolved taxonomic units are relatively prevalent (i.e., as compared to the median ASV prevalence) across multiple studies and facilities suggests that these ASVs should be experimentally prioritized for follow-up investigations of their physiological impacts as though they were core taxa. As noted in our results, these ASVs are members of *Aeromonas*, *Paucibacter*, *Flectobacillus*, *Vibrio*, *Shewanella*, the Comamonadaceae, and *Bosea.* These observations are at least in part consistent with prior work that resolved operational taxonomic units (OTUs) common to two different zebrafish facilities, as well as wild-caught fish [17]. In particular, this prior work discovered common OTUs that were members of *Aeromonas*, *Shewanella*, and *Vibrio* (as well as others not in our set of highly prevalent ASVs). Collectively, these observations suggest that specific strains of bacteria may exist which associate with zebrafish across facilities, possibly due to an intimate physiological association between these microbiota and their host. 

Our goal was to identify a robust core microbiome associated with healthy fish. To do so, we investigated zebrafish microbiome samples collected from the control (i.e., unexposed) arms of a variety of zebrafish studies. While this selection of samples affords an opportunity to identify a core microbiome that is robust to experimental and facility effects, it is imperfect for at least two reasons. Firstly, we do not possess direct insight into the physiology of these fish, so it is possible that these fish manifest cryptic diseases. However, given that the facilities that managed these fish provide extensive oversight of their husbandry and that these control arm fish were intended to represent healthy individuals in the investigations we selected, we presume our assumption of health is meaningful. Secondly, the control arm fish were not identical across all studies. In particular, they varied by strain, diet, tank conditions (e.g., recirculating versus flow through), water quality, and whether the fish were exposed to a control vehicle or not. As a result, more core taxa may actually exist than those which are uncovered here, because our investigation incorporated additional variation that may impact the core microbiome that associates with specific zebrafish strains, diets, or other related covariates. 

Future work should consider if and how core zebrafish gut microbiota affect zebrafish physiology and whether exposure-induced changes to these microbiota mediate how exposure impacts the host. For example, many of the core taxa identified in our investigation may actually represent taxa that are common to and abundant within zebrafish facilities (e.g., the tank microbiome). Such taxa may appear prevalent in the gut of zebrafish simply as a result of neutral dispersal processes rather than any sort of selection that would indicate functional effects on the host. On the other hand, common facility microbes may be selected for various physiological processes, such as intestinal motility [36], possibly because such microbes confer important functional effects on the host. Exposure to environmental toxicants that interfere with the success of these core taxa in the gut could manifest as toxicity. Our results can help future studies prioritize which microbiota should be functionally characterized in greater detail. 

Additionally, future studies should consider conducting similar integrations as those described here to better understand how different exposure conditions, study and facility designs, and fish genetics impacts the core gut microbiome. To help facilitate this work, we recommend that studies that generate zebrafish gut microbiome data ensure that their data and study covariates, especially physiological measures, are appropriately deposited into public data repositories. As the number of zebrafish fish microbiome studies continues to grow, such publicly available data will help to transform our understanding of the robustness of the results produced in this important model system.

## Figures and Tables

**Table 1:** Details of studies included in this analysis.

Lead Author	Year	Facility ^¥^	Number of Individuals	Age (dpf)	Exposure Conditions	SRA Bioproject Identifier	Citation
Catron	2018	EPA	118	10	Bisphenol A or related metabolites ^€^	NA *	[6]
Gaulke	2016	SARL	45	270	Triclosan	SRP071910	[2]
Gaulke	2019	OSU ZPV	210	127–213	*P. tomentosa*	SRA708553	[7]
Ma	2018	CAS	132	120	Silver nanoparticles	PRJNA348716	[5]
Phelps	2017	EPA	32	10	N/A	NA *	[10]
Pindling	2018	Bard College	10	96	Subclinical streptomycin	SRP139123	[3]
Stagaman	2017	Huestis	198	9, 75	N/A	SRA527217	[19]
Weitekamp	2019	EPA	53	6, 10	Triclosan	NA *	[4]

^¥^ EPA, The United State Environmental Protection Agency; SARL, Sinnhuber Aquatic Research Laboratory; OSU ZPV, Oregon State University Zebrafish Pathogenesis Vivarium; CAS, Chinese Academy of Science; Huestis, Huestis Zebrafish Facility at the University of Oregon. ^€^ Related metabolites are Bisphenol AF (BPAF), Bisphenol B (BPB), Bisphenol F (BPF) or Bisphenol S (BPS). * Received data directly from study authors.

## Data Availability

No new data were created in this study. Data was obtained from public databases or directly from study authors, as itemized in Table 1. Analytical results are available in Supplementary Table S1. Additional results or intermediate data files are available upon request from the corresponding author.

## Supplementary Materials

The following are available online at https://www.mdpi.com/2305-6304/9/1/10/s1, Table S1: Core Taxa Characteristics.
